# P-1497. Real-World 4CMenB Vaccine Duration of Protection in Infants and Children

**DOI:** 10.1093/ofid/ofaf695.1681

**Published:** 2026-01-11

**Authors:** Pavo Marijic, Iwona Zerda, Lucian Gaianu, Wojciech Margas, Katarzyna Jamroz-Dolinska, Thatiana Pinto, Gaurav Mathur, Anar Andani, Reena Ladak, Elise Kuylen, Helen Petousis-Harris, Zeki Kocaata

**Affiliations:** GSK, Munich, Bayern, Germany; Clever-Access, Kraków, Malopolskie, Poland; GSK, Munich, Bayern, Germany; Clever-Access, Kraków, Malopolskie, Poland; Clever-Access, Kraków, Malopolskie, Poland; GSK, Munich, Bayern, Germany; GSK, Philadelphia, Pennsylvania, USA, Philadelphia, Pennsylvania; GSK, Munich, Bayern, Germany; GSK, Philadelphia, Pennsylvania, USA, Philadelphia, Pennsylvania; GSK, Munich, Bayern, Germany; The University of Auckland, Auckland, Auckland, New Zealand; GSK, Munich, Bayern, Germany

## Abstract

**Background:**

Invasive meningococcal disease (IMD) is a devastating disease, with most cases caused by meningococcal serogroup B (MenB) in infants and children. The multicomponent MenB vaccine (4CMenB) has already shown high real-world effectiveness in various regions. Understanding the vaccine’s duration of protection (DoP) is crucial for determining the need for boosters, ensuring long-term immunity, and protecting populations from this disease. The purpose of this analysis was to explore the DoP of 4CMenB in fully vaccinated infants and children based on real-world vaccine effectiveness (VE) data.

**Table 1.**
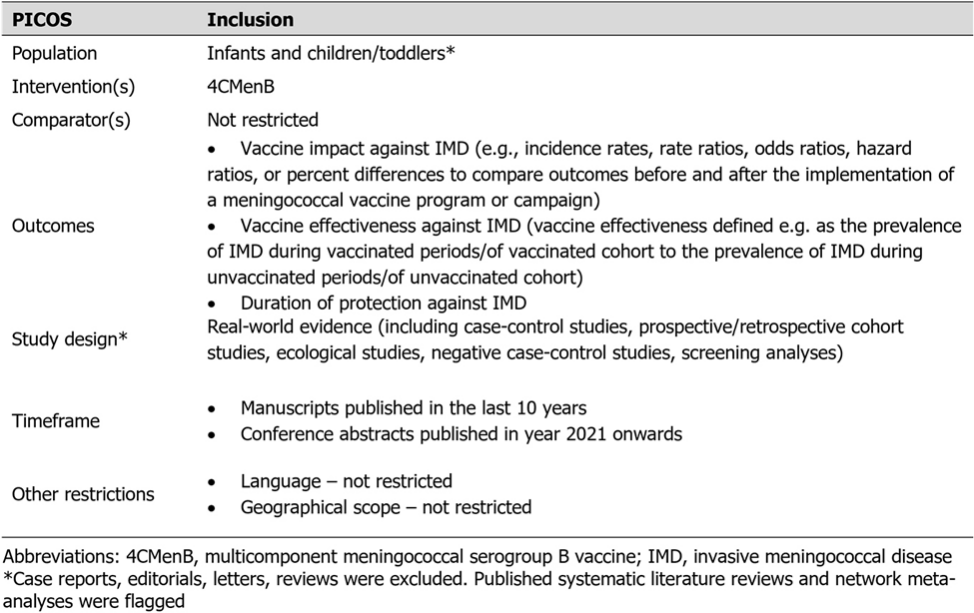
PICOS Criteria

**Figure 1.**
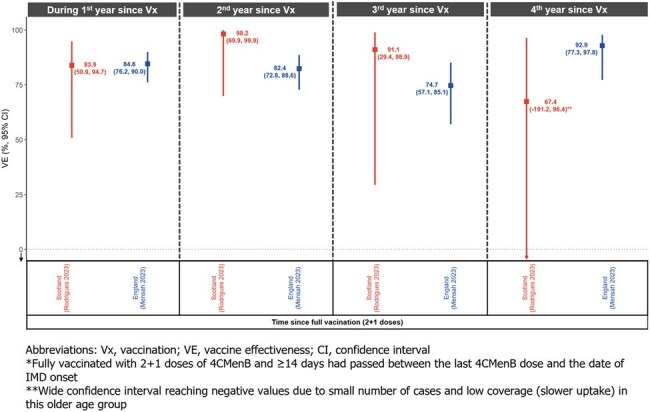
Vaccine Effectiveness Over Time in Fully Vaccinated* Infants and Children

**Methods:**

A systematic literature review (SLR) was conducted per PRISMA guidelines. MEDLINE, Embase, gray literature, and clinical trial registries were searched using predetermined criteria (Table 1). Fully vaccinated infants and children were defined based on vaccination schedules specific to each region or study. VE data were extracted for each year of age for studies reporting data up to 4 years post-vaccination. Where age-specific VE was not reported, the screening method was used when feasible to calculate VE based on information on cases in included studies and vaccination coverage data from publicly available sources, enabling the observation of VE over time and the inference of DoP.

**Results:**

Two studies from two regions (England and Scotland) identified in the SLR were eligible for this analysis. A further eligible study from Italy was excluded due to low case numbers that yielded highly uncertain estimates. VE was estimated for periods in 1-year increments up to 4 years since full vaccination.

Following a 2+1 dosing schedule, VE (95% confidence interval) ranged between 92.9% (77.3–97.8) and 74.7% (57.1–85.1) during the first 4 years since full vaccination in England, and between 98.2% (69.9–99.9) and 67.4.% (−191.2–96.4) in Scotland.

Considering the sustained high VE observed in England and Scotland during the first 4 years following full vaccination (Figure 1), a 2+1 dosing schedule of 4CMenB in infants provides a DoP of ≥ 48 months.

**Conclusion:**

4CMenB demonstrates sustained effectiveness for at least 48 months after the completion of the vaccination schedule in infants and children, providing durable protection for this age group that is most at risk for MenB-IMD.

Funding: GSK VEO-001056

**Disclosures:**

Pavo Marijic, PhD, GSK: employee|GSK: Stocks/Bonds (Public Company) Iwona Zerda, MSc, GSK: Collaborator with Clever-Access, which was paid by GSK to conduct this study Lucian Gaianu, MSc, GSK: Employee Wojciech Margas, PhD, GSK: Employee of Clever-Access, which was paid by GSK to conduct this study Katarzyna Jamroz-Dolinska, PhD, GSK: Employee of Clever-Access, which was paid by GSK to conduct this study Thatiana Pinto, PhD, GSK: employee|GSK: Stocks/Bonds (Public Company) Gaurav Mathur, MD, GSK: Employee|GSK: Stocks/Bonds (Public Company) Anar Andani, BSc, Medical director, GSK: Employee|GSK: Stocks/Bonds (Public Company) Reena Ladak, MS, GSK: Employee Elise Kuylen, PhD, GSK: Employee|GSK: Stocks/Bonds (Public Company) Helen Petousis-Harris, PhD, Bexsero and gonorrhea trial (USA): Data and Safety Monitory Board Member|CDC: Funding to institution|GSK: Advisor/Consultant|GSK: Funding to institution; payment for study|Maternal pneumococcal vaccine trial (Australia): Data and Safety Monitory Board Member|New Zealand Medical Council: Advisor/Consultant|The Ministry of Health New Zealand: Funding to institution Zeki Kocaata, PhD, GSK: Employee|GSK: Stocks/Bonds (Public Company)

